# Dual-In/Out strategy for genes integration into bacterial chromosome: a novel approach to step-by-step construction of plasmid-less marker-less recombinant *E. coli *strains with predesigned genome structure

**DOI:** 10.1186/1472-6750-8-63

**Published:** 2008-08-12

**Authors:** Natalia I Minaeva, Evgeny R Gak, Danila V Zimenkov, Aleksandra Yu Skorokhodova, Irina V Biryukova, Sergey V Mashko

**Affiliations:** 1 Closed Joint-Stock Company "Ajinomoto-Genetika Research Institute", 1st Dorozhny Pr. 1, Moscow 117545, Russia

## Abstract

**Background:**

The development of modern producer strains with metabolically engineered pathways poses special problems that often require manipulating many genes and expressing them individually at different levels or under separate regulatory controls. The construction of plasmid-less marker-less strains has many advantages for the further practical exploitation of these bacteria in industry. Such producer strains are usually constructed by sequential chromosome modifications including deletions and integration of genetic material. For these purposes complex methods based on *in vitro *and *in vivo *recombination processes have been developed.

**Results:**

Here, we describe the new scheme of insertion of the foreign DNA for step-by-step construction of plasmid-less marker-less recombinant *E. coli *strains with chromosome structure designed in advance. This strategy, entitled as Dual-In/Out, based on the initial Red-driven insertion of artificial φ80-*attB *sites into desired points of the chromosome followed by two site-specific recombination processes: first, the φ80 system is used for integration of the recombinant DNA based on selective marker-carrier conditionally-replicated plasmid with φ80-*attP*-site, and second, the λ system is used for excision of inserted vector part, including the plasmid *ori*-replication and the marker, flanked by λ-*attL/R*-sites.

**Conclusion:**

The developed Dual-In/Out strategy is a rather straightforward, but convenient combination of previously developed recombination methods: phages site-specific and general Red/ET-mediated. This new approach allows us to detail the design of future recombinant marker-less strains, carrying, in particular, rather large artificial insertions that could be difficult to introduce by usually used PCR-based Recombineering procedure. The developed strategy is simple and could be particularly useful for construction of strains for the biotechnological industry.

## Background

*Escherichia coli *is widely used in fundamental investigations and in modern biotechnology for production of biologically active compounds such as recombinant proteins, amino acids, vitamins *etc*. Construction of plasmid-less marker-less strains has advantages for extending the practical exploitation of these bacteria in industry [1-5]. Such producer strains are usually constructed by sequential chromosome modifications, mainly including deletions and integration of genetic material. For gene deletions, the Red/RecET recombination method developed by several groups [6-11] is considered as the most useful now. This method has been named Recombinogenic Engineering or Recombineering and reviewed in several papers [12-15]. It usually (but not always) based on λRed- or RecET-mediated recombination between bacterial chromosome and amplified DNA fragment carrying the removable selective marker, in which PCR primers provide the rather short homology to the targeted sequence. The integrated marker could be excised out of the chromosome by site-specific recombination. The Recombineering approach based on generated PCR products might be used not only for target genes disruption, but for integration of relatively short DNA fragments, for example, for substitution of recombinant regulatory regions (i.e. promoter, RBS) of a particular gene for its native regulatory region [16-19]. Although the special modifications of Recombineering procedure have been already developed for integration of large DNA fragments carrying several genes/operons (see, [20], for instance), more often special tools based on modified transposons [1,21-23], non-replicative [3] or conditionally-replicative plasmids [4,24-28] are using for the same purposes. Different transposons and phage Mu systems are exploited for introduction of the DNA cassettes into random points of the bacterial chromosome [1,21,29]. Integration into the native coliphages *attB *sites [30] or into artificially inserted recombinogenic sequences [3] is based on exploitation of corresponding site-specific recombination systems. By using cloned fragments of chromosomes as so-called "guides" [4] it is possible to integrate the cassette by general homologous recombination. In addition, combinations of different systems in one integration strategy are also used. For example, site-specific insertion of cassettes can be carried out in preliminary randomly integrated recombinogenic sites [3], or the "marked" cassettes can be randomly integrated, followed by excision of the marker by a site-specific recombination system [1].

**Figure 1:**
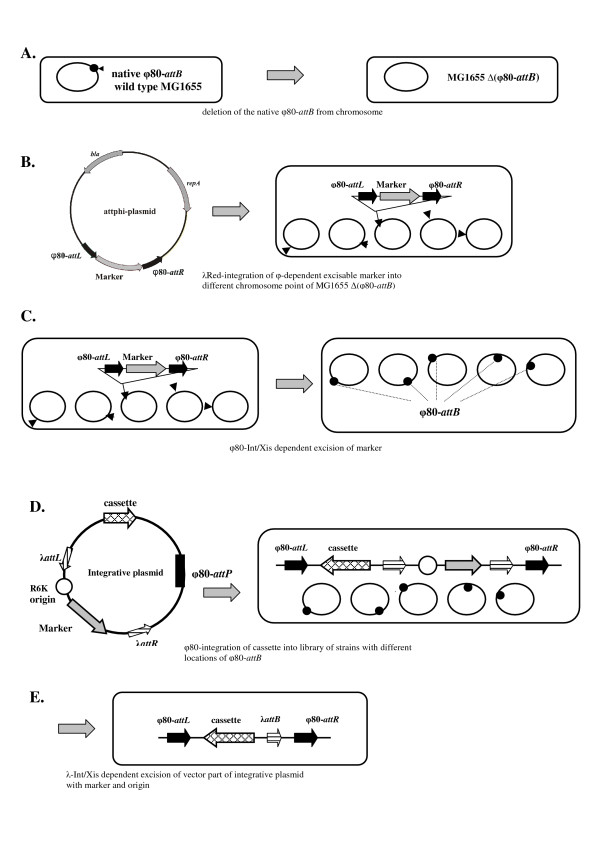
**Dual In/Out method for plasmid-less marker-less strain construction**. A: deletion of the native φ80-*attB *by Red recombination. B: Red integration of the φ80-removable marker to the desired loci of MG-Δ(φ80-*attB*) chromosome. C: curing of the marker by φ80-Int/Xis-system. D: φ80-driven integration of the CRIM plasmid with target cassette into the different of φ80-*attB *sites. E: construction of "marker-less" cassette-carrier-strains by λ-Int/Xis excision of the vector part of integrative plasmids.

For expansion of this group of methods, we propose a new strategy of DNA fragments integration in the process of plasmid-less marker-less recombinant *E. coli *strain construction. It initiates from Red-driven insertion of the antibiotic resistance marker flanked by φ80-*attL/R *sites, into the desired point of bacterial chromosome (this part of the total procedure is named as – the first "In") followed by φ80-Int/Xis-mediated excision of the marker (the first "Out) with retaining of φ80-*attB*-site. After that two sequential site-specific recombination processes are provided: first, the φ80-Int is used for integration (the second – "In") of the recombinant DNA constructed on the basis of conditionally-replicated plasmid with φ80-*attP*-site, and second, the λ-Int/Xis system [1,10,19] is used for excision of inserted vector part, including the plasmid *ori*-replication and the selective marker, that flanked by λ-*attL/R*-sites (the second – "Out"). So, the new strategy schematically presented in Fig. 1, could be named Dual-In/Out.

## Results

### Construction of strains with different locations of the φ80-*attB *site

The native φ80-*attB*-site is located at ~28 min of *E. coli *MG1655 chromosome ( and ). Insertion of the artificial φ80-*attB *into the chromosome of strain with deleted φ80-*attB *native site will allow us to introduce the DNA cassette into the new loci by φ80-Int-dependent system. If cassettes, integrated in the set of strains in different points, possess excisable selective markers, it will be possible to bring them in one strain by P1vir-mediated generalized transduction (P1-duction) due to removing the marker from the recipient genome before the next step of transduction.

The desired locations of φ80-*attB *sites can be spread in non-essential parts of bacterial genome at different distances from *oriC*. In this case, the level of expression of the same integrated cassette will depend on its position due to the θ-like structure of replicating chromosome and gene-dosage effect: the cassettes closer to *oriC *will be expressed at higher level than those located near the terminus of the chromosome replication [31].

We consider as good candidates for the further integration points the positions of the "native" IS elements (in our case IS5.7–IS5.11) since insertions into the genes already disrupted by IS elements will not be detrimental to cell viability and IS elements are almost randomly distributed along the chromosome of MG1655 [32]. Accordingly, the set of genes disrupted by IS5-elements insertion was chosen as the future locations of the artificial φ80-*attB *sites. These points are rather far from each other, so the future integrated cassettes can be combined in one strain by independent acts of P1-duction.

Realization of this strategy includes several steps. At first, the deletion of the native φ80-*attB *was carried out by Recombineering between *E. coli *MG1655 chromosome and the constructed "λ-excisable" Cm^R^-marker amplified by PCR from pMW118-(λ*attL- *Cm^R^-λ*attR*) [19]. After Red recombination, the DNA locus modification was verified by PCR, and followed by λ-Int/Xis-mediated excision of the marker from the chromosome. The marker-less strain, MG-Δ(φ80-*attB*), was used as the recipient for the insertion of the artificial φ80-*attB*. The latter includes:

1) construction and cloning of the cassette [(φ80-*attL*) - Km^R ^- (φ80-*attR*)] in the plasmid;

2) using the obtained plasmid, pMWattphi, as the template for PCR amplification of φ80-removable Km^R^-marker flanked by the 36 bp arms homologous to the desired loci on the MG1655 chromosome;

3) Red integration of the markers into the chromosome of MG-Δ(φ80-*attB*) for construction of the desired library of "marked" strains;

4) each obtained strain was cured from the marker by φ80-Int/Xis-system [30]. In this way the "unmarked" part of the library was constructed;

5) new strains and MG1655 (as a control) were tested for φ80-driven integration/excision of the "conditionally-replicated integrative and modular (CRIM) plasmid" pAH162 carrying φ80-*attP *site [30], using φ80-Int- or φ80-Int/Xis-helper plasmids, respectively. Here, the efficiencies of integration/excision In or Out of the artificial points were practically the same as for In/Out of the native site.

Although the efficiency of CRIM plasmids excision from all tested sites in our experiments was 15–30% (not 100%, as reported in [30]), nevertheless, selection the marker-less clones was not the problem. We noticed, as well that, in our hands, the efficiency of λ-Int/Xis-driven excision under the same conditions was significantly higher and exceeded 80%.

### Construction of φ80-integrative CRIM plasmid with λ-removable "vector part"

**Figure 2:**
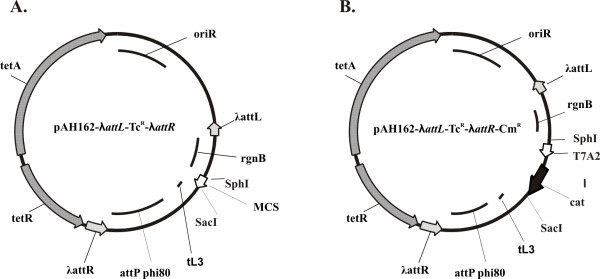
**New φ80-cognate CRIM plasmids with λ-removable part**. A. Map of pAH162-λ*attL*-Tc^R^-λ*attR*. This plasmid could be used as a vector for molecular cloning of the genes of interest followed by φ80-Int-dependent integration of the recombinant plasmid in bacterial chromosome and λ-Int/Xis-dependent excision of the selective marker-carrier vector part. B. Map of pAH162-λ*attL*-Tc^R^-λ*attR*-Cm^R^. The recombinant plasmid constructed on the basis of new CRIM-vector, that contains *cat-*gene under the transcriptional control of phage T7 A2-promoter, as a model gene for integration in the chromosome according to Dual-In/Out strategy.

The φ80-cognate CRIM plasmids with different selective markers can be used for integration of cassettes in the obtained strains that differ in location of φ80-*attB*. A recombinant strain that contains multiple insertions can be constructed by P1 transduction. However the presence of plasmids' markers in the bacterial chromosome cannot satisfy "marker-less" criteria for the practical application of the engineered strain. CRIM plasmids constructed by Haldimann and Wanner [30], only allow the site-specific excision of the entire recombinant structure initially inserted in the chromosome. It is useful to modify CRIM plasmids to allow excising of the vector part after site-specific integration of recombinant DNA. In this way, the new φ80-cognate CRIM plasmid (Fig. 2) with a λ-removable vector part was obtained (for details see Methods).

The new plasmid pAH162-λ*attL*-Tc^R^-λ*attR *(Fig. 2) retains from its progenitor pAH162 the non-modified fragment carrying φ80-*attP *and the multi-cloning site flanked by bacterial (*rgnB*) and phage λ (*tL3*) transcription terminators. This fragment is bracketed by λ-*attL/R *sites which allow λ-Int/Xis excision of the vector part, including conditionally-replicative origin of R6K (oriRγ) and the selective marker, Tc^R ^(from Tn10). We have confirmed the expected properties of pAH162-λ*attL*-Tc^R^-λ*attR *by its integration into the native φ80-*attB *of MG1655 chromosome, followed by λ-Int/Xis excision of the marked vector part flanked by λ-*attL/attR*. Of 80 tested clones cures from λ-Int/Xis-helper plasmid, more than 80% have lost the Tc^R ^marker of integrated CRIM plasmid, as well.

### Exploiting the new system for multiple integration of cat-gene into *E. coli *chromosome

To test the Dual-In/Out strategy of marker-less strain construction, we used the efficiently expressed variant of *cat*-gene as the model cassette. This experiment includes several steps:

1) cloning of *cat*-gene into pAH162-λ*attL*-Tc^R^-λ*attR*, using *E. coli *CC118 (λ*pir*^+^) strain as the recipient;

2) φ80-driven integration of the recombinant CRIM plasmid into the strains that differ in location of φ80-*attB*;

3) construction of "marker-less" strains with single *cat*-cassette by λ-Int/Xis excision of Tc^R^-containing DNA fragment flanked by λ-*attL/R*-sites, followed by determination of *cat *expression levels in these strains;

4) combining the set of *cat*-cassettes in one "marker-less" strain by sequential P1-duction of the "marked" cassettes followed by curing the Tc^R^-marker from the recipient strain before the next stage of transduction.

The previously constructed [19] pMW118-(λ*attL*-Cm^R^-λ*attR*) plasmid was chosen as a template for PCR amplification of *cat*-gene. In this plasmid the structural part of *cat*-gene with its native RBS from *E. coli *Tn9 is under the transcriptional control of rather strong phage T7 A2 promoter [33] that is recognized by *E. coli *RNA polymerase with σ70 in a constitutive manner. This *cat*-gene was amplified by PCR with the primers that carry restriction sites for cloning in pAH162-λ*attL*-Tc^R^-λ*attR*. The transformants of *E. coli *CC118 (λ*pir*^+^) carrying the recombinant plasmid of interest, were selected on the medium supplemented with Cm. The expected structure of the plasmid was verified by restriction analysis and PCR.

**Figure 3:**
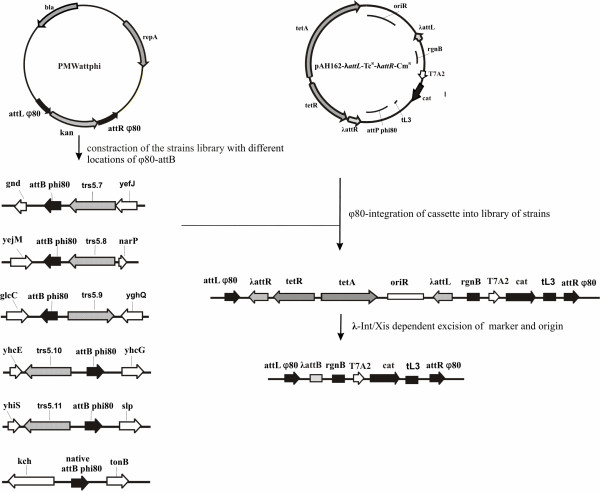
Integration of plasmid pAH162-λ*attL*-Tc^R^-λ*attR*-Cm^R ^into MG1655-derived strains with different location of φ80-*attB*.

At the next stage this plasmid was integrated by φ80-Int system into MG1655-derived strains with a different location of φ80-*attB*, using Tc^R^-marker for selection (Fig. 3). The correct integration was confirmed by PCR, and the corresponding strains were entitled MG-Tc^R^-*cat*-(i), where (i) varied from 1 to 6 depending on the number of φ80-*attB *sites. Subsequently, the vector part of the integrated plasmids in all MG-Tc^R^-*cat*-(i) strains was excised by λ-Int/Xis site-specific recombination. After checking by PCR, the corresponding recombinant strains retaining the *cat*-gene in their chromosomes, was entitled MG-*cat*-(i).

**Figure 4:**
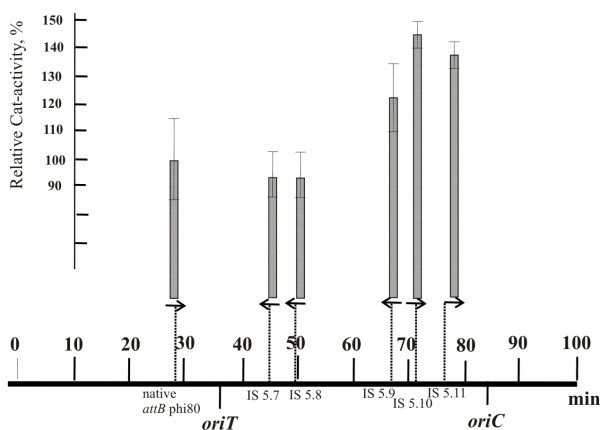
**The *cat*-gene expression in MG-*cat*-(i)-strains**. The dependence of the expression level of the *cat*-carrier cassetes (T7A2-*cat*) on their positions in the chromosome is indicated by arrows. Cat-activity of the MG-*cat*-(6) strain in which Cassette (T7A2-*cat*) is located in native φ80-*attB *was taken as 100%.

The efficiency of *cat*-gene expression in MG-*cat*-(i)-strains was evaluated by determination of Cat enzymatic activity (Fig. 4, Table 1). As expected (see explanation above and reference [31]), the level of *cat*-gene expression correlates with the distance between the integration point and *oriC*: the strains with the *cat*-gene position closer to *oriC *have higher Cat activity.

**Table 1:** Cat-activity of tested MG-*cat*-(i) and MG-*cat*-(i+j) strains. The results were averaged over three independently grown cultures for each clone; the scatter was 5–15%.

**strain MG-*cat*-(i)**	**IS element**	**Activity, nmol/minxmg**
MG-*cat*-(1)	IS 5.7	180 ± 15
MG-*cat*-(2)	IS 5.8	180 ± 15
MG-*cat*-(3)	IS 5.9	240 ± 30
MG-*cat*-(4)	IS 5.10	280 ± 15
MG-*cat*-(5)	IS 5.11	270 ± 15
MG-*cat*-(6)	native φ80-*attB*	200 ± 30
**MG-*cat-*(i+j)**		
MG-*cat*-(1+2)	IS 5.7 IS 5.8	330 ± 15
MG-*cat*-(1+3)	IS 5.7 IS 5.9	420 ± 30
MG-*cat*-(2+3)	IS 5.8 IS 5.9	420 ± 30
MG-*cat*-(1+2+3)	IS 5.7 IS 5.8 IS 5.9	520 ± 15
MG-*cat*-(4+5)	IS 5.10 IS 5.11	500 ± 30
MG-*cat*-(4+6)	IS 5.10 native φ80-*attB*	370 ± 15
MG-*cat*-(5+6)	IS 5.11 native φ80-*attB*	400 ± 15
MG-*cat*-(4+5+6)	IS 5.10 IS 5.11 native φ80-*attB*	540 ± 30

Several *cat*-cassettes were combined in one strain by sequential P1-duction of Tc-marked fragments from MG-Tc^R^-cat-(i) strains into MG-*cat*-(j) (where i ≠ j), followed by λ-Int/Xis-driven curing of Tc^R^-marker that gives an MG-*cat*-(i + j) "marker-less" strain carrying two *cat*-cassettes in (i) and (j) positions; a third cassette was added by an analogous procedure. In addition, all steps used in increasing the number of *cat*-cassettes in the chromosome of recombinant "marker-less" strain were controlled by determination of Cat activity. The data presented in Table 1 show that the total Cat activity of multi-integrants correlates with the expected sum of the Cat activities of the corresponding single-integrants.

## Discussion

In this paper we have described the development of a new strategy for the integration of genes/operons of interest during plasmid-less marker-less recombinant strain construction, which we have named Dual-In/Out. It combines Red-mediated insertion of the artificial φ80-*attB *site into desired point of bacterial genome, φ80-Int-dependent site-specific integration of recombinant DNA of interest constructed on the basis of specially constructed CRIM plasmid followed by λ-Int/Xis-mediated excision of the plasmid's vector part flanked by λ*attL/R*, out of the chromosome.

In this study a library composed of five strains that differ in the position of inserted φ80-removable Km^R^-marker and five isogenic marker-less strains for the possible φ80-dependent integration of a cassette has been obtained. (In fact, the sixth strain from this library is the MG1655 wild type with the native φ80-*attB *site). The "marked" part of this library can be used to select, in advance, the desirable points for the cassette integration that can be useful for construction (modification) of the producer strain. This is achieved by checking that transduction of the marker to a particular location does not affect the producer features (growth rate, desirable product yield, etc.). After preliminary selection of the members of the library based on testing "marked" strains, the corresponding "unmarked" variants can be directly used for the φ80-driven integration of cassette(s).

This library can be extended by the Red-mediated insertion of φ80-*attB *sites into new points on MG1655 chromosome with deleted native φ80-*attB*. The plasmid pMWattphi can be used as the template for PCR with the primers for this purpose. The designing of the primers can be based on the known "native" insertions in the *E. coli *genome, as described in this paper. On the other hand, φ80-*attB *integration can be used for simultaneous inactivation of an "undesirable" gene whose expression decreases the performance of a producer strain. It is important to keep in mind that the integrated cassette "ter *rgnB *– (genes of interest) – *tL3*" could be downstream from a native transcription unit in the chromosome. In this case, terminator *tL3 *prevents transcription of chromosomal genes originating in the cassette, and ter *rgnB *protects the cassette from the bacterial transcription read through.

In earlier techniques, the library of *E. coli *strains with different positions for site-specific (Flp-dependent) integration was constructed by random insertions of corresponding recombinogenic (FRT) sites by the Tn5-system [3]. At first glance, this library may have the same applications as the library described in this paper. Moreover, its improvement requires only determination of the FRT integration points for already obtained several hundreds of independent Tn5-driven integrants [3]. On the other hand, the expansion of our library depends on the separate Red-driven integrations, each leading to construction of only one new member. However, Red-driven integration is now used as a routine procedure and, according to our experience, can be provided in a quantity of several tens per week. At the same time our library has two advantages: (i) due to the initially designed points of the φ80-*attB *insertion we can exclude the interference between sequentially introduced cassettes, while random insertions more often localize near *oriC *due to a gene dosage effect produced by replication of the bacterial chromosome [29]; (ii) it is possible to combine distribution of site-specific integration points simultaneously with deletion of some "undesirable" genes.

As already mentioned, the Dual-In/Out strategy allows construction of marker-less strain carrying different cassettes and/or several copies of the same cassette. The presence of several copies might be necessary to increase the level of the cassette expression. However, transduction of additional copies into the same strain may lead to possible chromosome rearrangements due to general recombination events between repeated sequences.

These questions of general recombination can be divided in two groups. The first one is the question of the additional insertion of the new "marked" cassette during the transduction process, not into the point corresponding to its location in the donor genome, but into the "unmarked" cassette in the recipient chromosome. This will lead to substitution of the new cassette for the previous one. Theoretically, this process can not be excluded, but its probability is extremely low due to the small distance between φ80-*attP *and λ-*attR *in the plasmid pAH162-λ*attL*-Tc^R^-λ*attR *which is used as the vector for the cassettes cloning (Fig. 2), and so, the size of the possible "arm" for envisaged general recombination would be too small. At least, we have never detected substitutions instead of expected amplification in tested clones. In any case, verifying the amplification of the integrated cassettes by PCR is desirable.

Concerning the stability of maintaining identical cassettes in the genome, direct or inverted repeats should be considered. In case of directly repeated cassettes the general recombination between them would lead to the deletion of part of the bacterial genome. According to the proposed design of the integration points, the distance between the cassettes will exceed the size of the transducing phage genome (about 100 kb in case of P1 phage). Only a few parts of the *E. coli *MG1655 chromosome more than 100 kb in length, do not contain any of the ca. 230 absolutely essential genes. If the designed points of integration are outside these few parts, the strains with the deleted regions between directly repeated Cassettes will not survive. General recombination between long inverted repeats leads to the chromosomal inversions that, in turn, can change the properties of the strain [34-36]. Although these events are rather rare, it could be recommended to avoid construction of the strains with inversely repeated copies of the cassette. This is not a difficult task, because the developed Dual-In/Out strategy of strain construction allows the determination, in advance, not only of location, but also of the orientation of the desired insertions.

## Conclusion

Summing up, the developed Dual-In/Out strategy is rather straightforward, but convenient combination of previously developed methods. Previous approaches of integration of rather large DNA fragments, usually, use only one high-performance site-specific recombination system. When the site-specific recombination is used for insertion of the fragment, the selective marker remains in the chromosome [30]. When it is used for excision of the selective marker, the initial integration of the cassette is carried out by general recombination [3]. The combination of Red/ET-driven and two site-specific recombination systems in one strategy for integration cassettes carrying several genes/operons, during construction of marker-less strains with desired structure is rather obvious, and, probably, it will be useful for fundamental and applied fields of microbiology and biotechnology.

## Methods

### Strains and plasmids

*Escherichia coli *K-12 MG1655, a wild-type strain with sequenced genome [32], was used as the recipient for the insertion of the artificial φ80-*attB*. CRIM plasmids were propagated in CC118 λ*pir*^+ ^strain [37]. *E. coli *W3350(80) lysogenic for phage φ80 was obtained from GosNIIGenetika collection and used as a template for PCR-driven amplification of φ80-*attL/R *sites.

pKD46 was used as a donor of λRed-genes for providing Red-dependent recombination according to the described procedure [8].

pAH123 and pAH129-helper plasmids [30], GenBank accession numbers AY048726 and AY048727 respectively. These helper plasmids were used for φ80-dependent integration/excision procedures.

pMWts-λInt/Xis-helper plasmid is similar to the plasmid pMP955A described in [1]. It has low-copy-number thermo-sensitive replicon pSC101, genes *xis *and *int *of phage λ under the control of λP_R_, thermo-sensitive repressor *cI*ts857. It is used for λ-Int/Xis-mediated excision of the DNA fragments flanked by λ*attL/R*.

pMWattphi – this recombinant plasmid was constructed on the base of pMW118 (GenBank accession number AB005475). This plasmid is used as the template for PCR amplification of fragment (φ80-*attL*) - Km^R ^- (φ80-*attR*) flanked with 36 bp arms homologous to targeted site in MG1655 DNA. Hybrid φ80-*attL *and φ80-*attR *sites were obtained by PCR amplification from purified chromosome of *E. coli *W3350(80) using primers: P1 – P2 for *attL *and P3 – P4 for *attR*. Amplified fragments were restricted with EcoRI-BamHI and XbaI-PstI restrictases and cloned into corresponding sites flanking *kan *on plasmid to give pMWattphi (Fig. 3).

P1 5'-atagaattcgaaaggtcatttttcctgaatatgc-3'

P2 5'-ataggatccatcattgaatgggtacacatttttg-3'

P3 5'-atattctagagatttgaatagcgagcgtaccttag-3'

P4 5'-atactgcagtcgtttgttgacagctggtccaatg-3'

pAH162-λ*attL*-Tc^R^-λ*attR *– integrative plasmid. Construction of this plasmid included several steps with isolation and analysis of recombinant DNA intermediates. The structure of this plasmid is shown in Fig. 2. Sequence landmarks: 1) from 6 to 1031 – fragment from pAH162 (GenBank accession number AY048738) which contains conditional-replication origin oriRγ; 2) from 1038 to1145 – fragment contains *attL *of phage λ from plasmid pMW118-(λ*attL*-*tetA-tetR*-λ*attR*) which structural similar to the plasmid pMW118-(λ*attL*-Cm^R^-λ*attR*) [19]; 3) from 1153 to 2274 – fragment from pAH162 which contains bacterial terminator *rgnB*, multiple cloning sites MCS, phage λ terminator *tL3*, phage attachment *attP *phi80; 4) from 2281 to 2462 – fragment contains *attR *of phage λ from plasmid pMW118-(λ*attL-tetA-tetR*-λ*attR*); 5) from 2456 to 4463 – fragment from plasmid pMW118-(λ*attL-tetA-tetR*-λ*attR*) which contains the Tn10-encoded tetracycline resistance gene *tetA *and the repressor gene *tetR*.

pAH162-λ*attL*-Tc^R^-λ*attR*-Cm^R ^was constructed by cloning of the SphI-SacI *cat*-carrier DNA fragment amplified from pMW118-(λ*attL*-Cm^R^-λ*attR*) [19] with primers P5 – P6.

P5 5'-cagtaagcatgcgcggccgcccggataagtagacagcctgataag-3'

P6 5'-cagtaagagctcgcggccgcttacgccccgccctgccactc-3'

### Molecular biology methods

Restriction analysis of the recombinant plasmids and Ca^2+^-dependent transformation of *E. coli *cells were performed in accordance with the routine experimental protocols [38]. Commercially available preparations of restrictases, T4 DNA ligase and the Klenow fragment of *E. coli *DNA polymerase I (Fermentas, Lithuania) were used. PCR fragment for cloning were generated by using AccuTaq DNA polymerase (Sigma, USA). Sigma (USA) products were used for the isolation of plasmid DNA, extraction of DNA fragments from agarose gels.

### Construction of strains with different locations of the φ80-*attB *site

The deletion of the native φ80-*attB *was carried out by Red-dependent integration of "λ-excisable" Cm^R^-marker which has been amplified by PCR from pMW118-(λ*attL*-Cm^R^-λ*attR*) [19] with primers P7 – P8. DNA locus modification was verified by PCR with primers P9 – P10.

P7 5'-gtaatcaaaggatttgagcgagcaactgtacctcagcgctcaagttagtataaaaaagctgaac-3'

P8 5'-acatttagcacgtttacagttactgcatgatgaaggtgaagcctgcttttttatactaagttgg-3'

P9 5'-tgcagcgcgtgaatgtgtta-3'

P10 5'-ctcaagacaaagctgatagcc-3'

The obtained marker-less strain, MG-Δ(φ80-*attB*), was used as the recipient for the insertion of the artificial φ80-*attB*. pMWattphi was used as the template for PCR amplification of fragment (φ80-*attL*) - Km^R ^- (φ80-*attR*) which integrated into the chromosome of MG-Δ(φ80-*attB*) to the desired loci – the set of genes disrupted by IS5-elements insertion. For these purposes the following primers were used:

IS5.7 P11 5'-tcctaaagaaagtatctattctgatacggttgttgagaaaggtcatttttcctgaatatg-3'

P12 5'-aagccatttacacgcacaaaatctgaaaaacgtacctcgtttgttgacagctggtccaatg-3'

P13 5'-gtcttctcacgggaacggtt-3'

IS5.8 P14 5'-gagggtatcagtacattgaaatgaatggcgccgcaggaaaggtcatttttcctgaatatg-3'

P15 5'-tctggtttgccgcgccacccatttgaacaatttgattcgtttgttgacagctggtccaatg-3'

P16 5'-cctcccttttcgatagcgacaa-3'

IS5.9 P17 5'-gggcgtattaccgcgcaaatagataccttgcaccgcgaaaggtcatttttcctgaatatg-3'

P18 5'-ctgcggatcatcaatggcgtcaatcatgccgaaatg-tcgtttgttgacagctggtccaatg-3'

P19 5'-gttcaatatgcgcggcatacca-3'

IS5.10 P20 5'-tatcaattgacgttaaggtgactctggaagctgcaggaaaggtcatttttcctgaatatg-3'

P21 5'-tattgactgaatgactaccgaagttaacaactccgctcgtttgttgacagctggtccaatg-3'

P22 5'-ttccggtggtcatactatccattc-3'

IS5.11 P23 5'-attattaaccattaatgacaaccttttacgagcaaagaaaggtcatttttcctgaatatg-3'

P24 5'-tatgaaagattggttatcctggcctctaaaaatttatcgtttgttgacagctggtccaatg-3'

P25 5'-ctttttcattaggcagtggcctc-3'

The integration of fragment was verified by PCR with primers P13, 16 – P26 and P19, 22, 25 – P27.

P26 5'-tgtttcgggcggaccaaatgata-3'

P27 5'-gccatggcagaatctgctccatgcggg-3'

### Plasmid integration and excision of the vector part of integrated plasmid

For testing the new MG1655 strains with artificial φ80-*attB *sites and CRIM plasmid integration/excision, procedures were driven by standard protocols using helper plasmids pAH123 (φ80-Int) and pAH129 (φ80-Int/Xis) respectively [30]. The vector part of CRIM plasmids was excised by λInt/Xis – site-specific recombination using helper plasmids pMWts-λInt/Xis by standard protocols [1]. The integration of plasmid was verified by PCR with primers P13, 16, 19, 22, 25, 28 – P30 and P 26, 27, 29 – P31. P28 and P29 are primers for native φ80-*attB *[19]; P30 is a primer annealing on vector part of integrated plasmid [19] and P31 is annealing on *tetR *gene.

P28 5'-taaggcaagacgatcagg-3'

P29 5'-ctgcttgtggtggtgaat-3'

P30 5'-acgagtatcgagatggca-3'

P31 5'-gtaaactcgcccagaagctagg-3'

### Cat activity assays

The Cat activity was assayed using a spectrophotometric method (UVmini 1240; Shumadzu, Japan). Log-phase cells harvested at OD_595 _= 0.8 were resuspended in potassium phosphate buffer (50 mM; pH 7.5). Cell lysates were prepared by sonication. The quantity of protein was determined by the Bradford method [39]. Assays were performed in 1 ml (1 cm light path) cuvettes at room temperature. The reaction mixture in each cuvette contained 100 μl of 1 M Tris-hydrochloride, pH 7.5, 100 μl of 1 mM acetyl CoA (Sigma, USA), 100 μl of 10 mM 5, 5'-dithio-bis-2-nitrobenzoic acid (DTNB; Sigma, USA), 0.05 mg of protein, H_2_O for a total volume of 0.99 ml. 10 μl of 10 mM Cm (Sigma, USA) was added to start the reaction, and thionitrobenzoic acid (TNB) production was followed at 412 nm. Enzyme activity was calculated in terms of nmol of thionitrobenzoic acid produced per min per mg of protein. The results were averaged over three independently grown cultures for each clone; the scatter was no more than 15%.

## Abbreviations

PCR: polymerase chain reaction; Cm: chloramphenicol; Km: kanamycin; Tc: tetracyclin.

## Authors' contributions

NIM obtained the library of strains with different locations of the φ80-*attB *site, performed the integration of plasmid pAH162-λ*attL*-Tc^R^-λ*attR*-Cm^R ^to these strains, carried out Cat-assay experiments and edited the manuscript. ERG and DVZ designed the primers and the construction scheme and constructed pMWattphi and pAH162-λ*attL*-Tc^R^-λ*attR *plasmids. AYS and IVB participated in the design of the study and helped draft the manuscript. SVM supervised and coordinated the work and edited the manuscript. All authors read and approved the final manuscript.
